# Beyond the surface: a review of transcranial temporal interference stimulation for deep brain modulation

**DOI:** 10.3389/fneur.2025.1661049

**Published:** 2025-09-22

**Authors:** Boyan Ivanov, Jake E. Toth, Alekhya Mandali, Roberto Salvati, Mahnaz Arvaneh

**Affiliations:** ^1^School of Electrical and Electronic Engineering, The University of Sheffield, Sheffield, United Kingdom; ^2^Institute of Biomedical Engineering, The University of Oxford, Oxford, United Kingdom; ^3^School of Psychology, The University of Sheffield, Sheffield, United Kingdom; ^4^Perspectum Ltd., Oxford, United Kingdom

**Keywords:** non-invasive deep brain stimulation, temporal interference stimulation, interferential stimulation, interfering electric fields, temporal interference stimulation (TIS), interferential current stimulation, transcranial temporal interference stimulation (tTIS)

## Abstract

Temporal Interference (TI) stimulation has emerged as a novel, non-invasive technique for selectively modulating deep brain regions while minimizing stimulation of superficial cortical layers, addressing key limitations of traditional transcranial electrical stimulation (tES) methods. This review systematically examines advancements in TI research from June 2017 to December 2024, encompassing safety evaluations, computational modeling (including Finite Element Method simulations), and stimulation–parameter optimisation. The paper synthesizes 63 publications on the efficacy of TI in deep brain neuromodulation, its optimisation strategies, and emerging methodologies aimed at improving stimulation precision and reducing off-target effects. Furthermore, the review explores the clinical applications of TI, particularly its potential in treating neurological disorders such as epilepsy, Parkinson's disease, and cognitive impairments. Despite its promise, challenges remain, including variability in stimulation outcomes, the need for individualized treatment protocols, and gaps in understanding the long-term effects of TI. By consolidating current knowledge and identifying future research priorities, this review provides a comprehensive perspective on the transformative potential of TI stimulation in neuroscience and clinical neurotherapeutics.

## 1 Introduction

The applications of transcranial electrical stimulation (tES) toward restoring human health have been advancing rapidly in the last decade. However, standard tES delivers current through large sponge pads, where a recent review of optimisation studies notes that, even with multi-electrode configurations, targeting deep structures inevitably co-stimulates overlying cortex (1). Modeling work shows that much of the injected current is shunted across the scalp, producing diffuse fields that decay rapidly with depth (2), and practical guides warn that scalp electrodes do not effectively penetrate deep brain regions, making them most suitable for cortical targets (3). Temporal Interference (TI) stimulation, a technique developed by Grossman et al., has been shown to non-invasively target deep brain structures, opening new possibilities for therapeutic interventions in neurological disorders (4).

**Figure 1:**
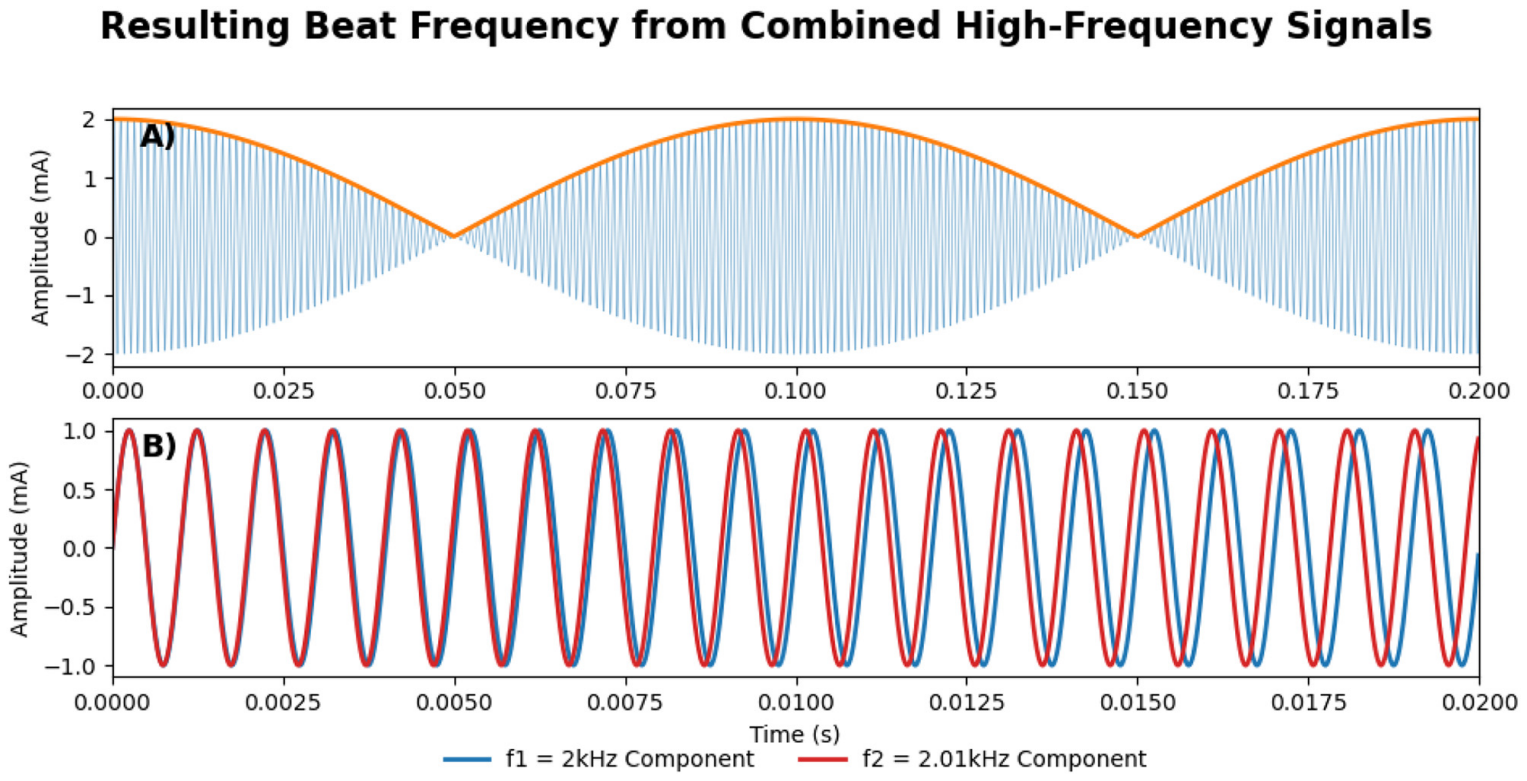
**(A)** The amplitude-modulated signal, resulting from the constructive interference between the two component high-frequency signals, where the orange line shows the resulting low-frequency envelope, oscillating at Δ*f* = *f*1−*f*2. **(B)** The two high-frequency component signals (Red and Blue), *f*1 and *f*2, are also shown in Figure 2. Please note that the scale in the x-axis between **(A, B)** is different.

**Figure 2:**
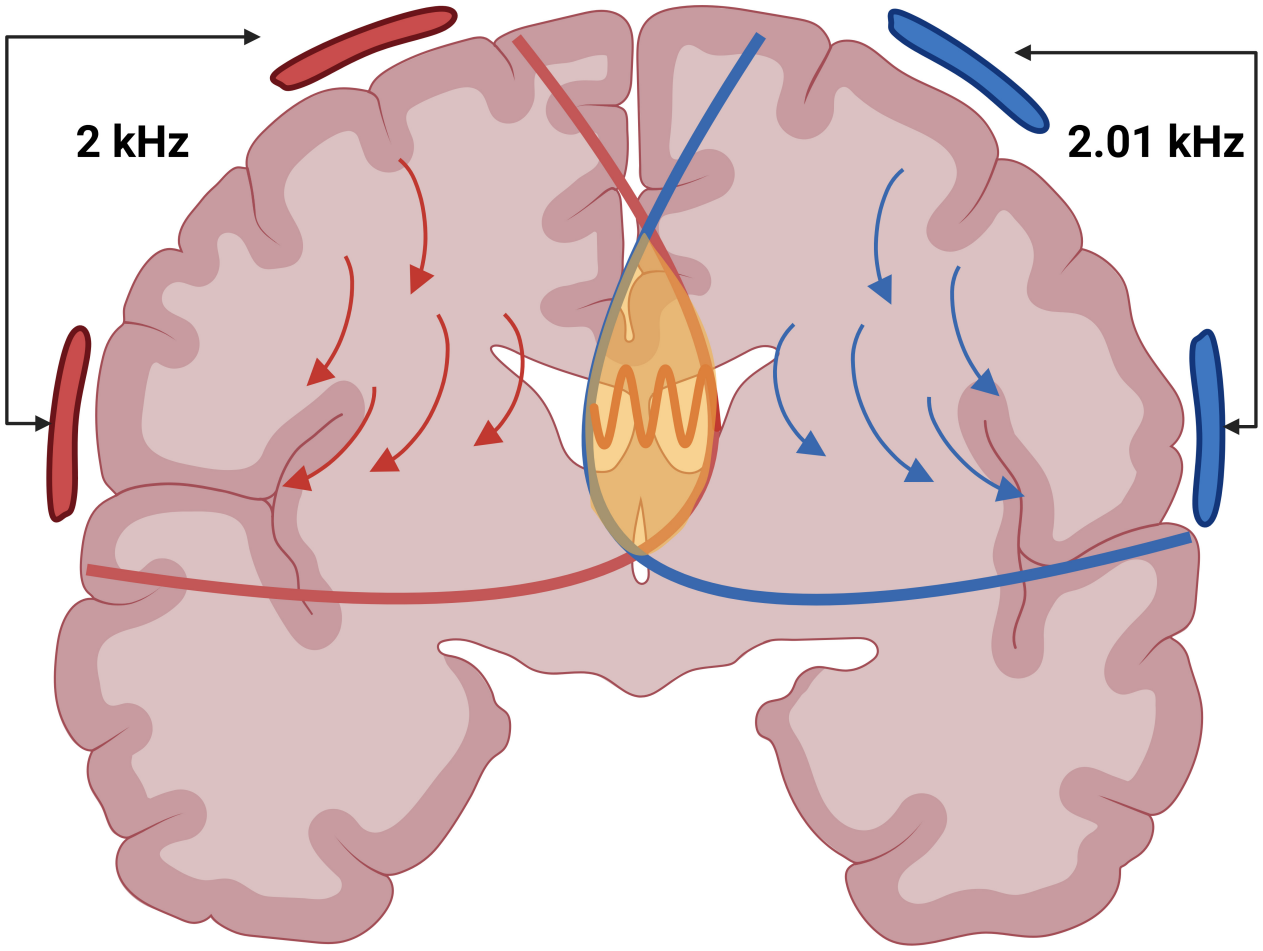
An illustration depicting two high-frequency electric fields in red and blue and the stimulation point in yellow where the Beat Frequency is formed. Created in BioRender. Ivanov, B. (2025) https://BioRender.com/jg6woc6.

TI stimulation applies two slightly different high-frequency alternating currents (*f*1 and *f*2) through two pairs of electrodes on the scalp. These currents interfere constructively and produce an electric field that oscillates at the difference of their frequencies (Δ*f* = *f*1−*f*2), called the beat frequency (see Figure 1). While neuronal membranes exhibit low-pass filtering properties at the level of subthreshold membrane responses (5, 6), While neuronal membranes exhibit low-pass filtering properties at the level of subthreshold membrane responses (5, 6), TI can effectively stimulate deep brain structures while largely avoiding direct neural activation of superficial cortex (7–9). However, overall current amplitude remains limited by cutaneous perceptibility and pain thresholds in scalp tissues (10). Although TI stimulation produces a low-frequency “beat,” all delivered energy remains confined to the high-frequency carriers. Neurones, acting as nonlinear rectifiers, demodulate the high-frequency carriers and respond at the beat rate (6), even though no low-frequency power is injected directly. By strategically placing electrode pairs, the targeted superimposed field is directed deep within the brain (see Figure 2). However, recent studies suggest that this understanding may be incomplete, as network-level interactions and ion channel non-linearities appear to play a crucial role in TI-induced neural responses (11, 12). While these findings challenge the conventional model, the precise mechanisms of action underlying TI stimulation remain uncertain and continue to be actively investigated.

In rodent models, TI has been shown to induce depolarisation of axonal membranes, potentially generating action potentials at the cellular level (4). However, in humans, TI functions as a sub-threshold modulation technique similar to transcranial alternating current stimulation (tACS) and transcranial direct current stimulation (tDCS). Some of the contributing factors for this difference are attributed to the larger brain size, increased skull thickness, and the lower current amplitudes applied. This allows for modulation of neural oscillations, similar in function to transcranial alternating current stimulation (tACS) and transcranial direct current stimulation (tDCS) (13). Through oscillation of the modulation envelope, TI can synchronize with existing neural activity via the principle of neural entrainment, reminiscent of tACS (14). By doing so, TI can influence neural behavior and connectivity without directly triggering action potentials in humans. This sub-threshold method enables TI to target deep brain structures—such as the hippocampus, striatum, and motor cortex—non-invasively. A recent study by Violante et al. (7) demonstrates TI's capacity to modulate the hippocampus in humans, presenting promising applications for both research and therapeutic interventions in regions traditionally difficult to reach with tES.

In TI stimulation, the depth and focality of the resulting electric field (e-field) are influenced by several critical factors, including the amplitude, frequency, phase, and spatial configuration of the applied currents (13, 15–18). Higher current amplitudes enable deeper brain penetration but may compromise focality, increasing the risk of unintended stimulation or conduction blocks (13, 19–21). The phase and frequency settings play a pivotal role in shaping the interference patterns, which directly affect the location and extent of the e-field (15). Additionally, the spatial arrangement and number of electrodes are crucial for focusing the e-field on specific target areas while minimizing stimulation of adjacent tissues (22). Optimizing these parameters is crucial for developing safe and effective TI protocols tailored to therapeutic and experimental needs.

## 2 Article types

Recent findings on TI stimulation remain promising but highlight significant knowledge gaps and methodological inconsistencies. While earlier reviews have covered aspects of the TI literature, they remain limited in scope. Zhu and Yin (87) highlighted inconsistencies in human outcomes and called for improved stimulation protocols, focusing on preclinical and computational studies. Demchenko et al. (23) reviewed human studies, emphasizing safety and early therapeutic outcomes, but omitted animal and computational research. Gomez-Tames et al. (1) discussed electric-field modeling for tES, with only peripheral coverage of TI. Soroushi et al. (24) explored computational strategies but lacked integration of clinical safety data. Peng et al. (25) mapped preclinical mechanisms without addressing human trials or safety. Key gaps remain in synthesizing data on safety and tolerability, optimizing stimulation parameters, integrating findings across animal, computational, and clinical domains, and linking preclinical insights to clinical protocols. Our scoping review addresses these by mapping safety data, modeling approaches (including finite element analysis), and parameter optimisation strategies across modalities. We reconcile fragmented methodologies and provide a unified foundation for advancing TI research and clinical translation.

Unlike systematic reviews or meta-analyses, which focus on narrowly defined questions and often impose strict inclusion criteria, a scoping review is designed to map the breadth of a developing research area, clarify key concepts, and identify knowledge gaps and research priorities (26, 27). Given the early-stage nature of TI studies—spanning human safety trials, animal models, and diverse computational simulations—a scoping methodology allows us to comprehensively chart existing work, accommodate multiple study designs, and survey areas requiring deeper, focused investigation.

This scoping review synthesizes existing research on TI safety, parameter optimisation, and computational simulations, encompassing both experimental and modeling studies. The objective is to identify effective strategies, common challenges, and future research priorities to advance the development of TI stimulation toward clinical adoption. By consolidating studies on TI's application in the human brain, this review highlights key trends, limitations, and knowledge gaps, along with recent advancements aimed at addressing these challenges.

The review begins with an overview of TI mechanics, followed by an evaluation of its safety and tolerability in humans. Next, it assesses TI's impact on neuronal activation and excitability through computational models, comparing its performance with traditional tES methods. Subsequently, optimisation methodologies and advanced techniques to enhance TI's efficacy are explored, along with novel modalities and their potential benefits. The review concludes by discussing the clinical applications of TI and its future potential in neurotherapeutics.

## 3 Literature search and inclusion/exclusion criteria

**Figure 3:**
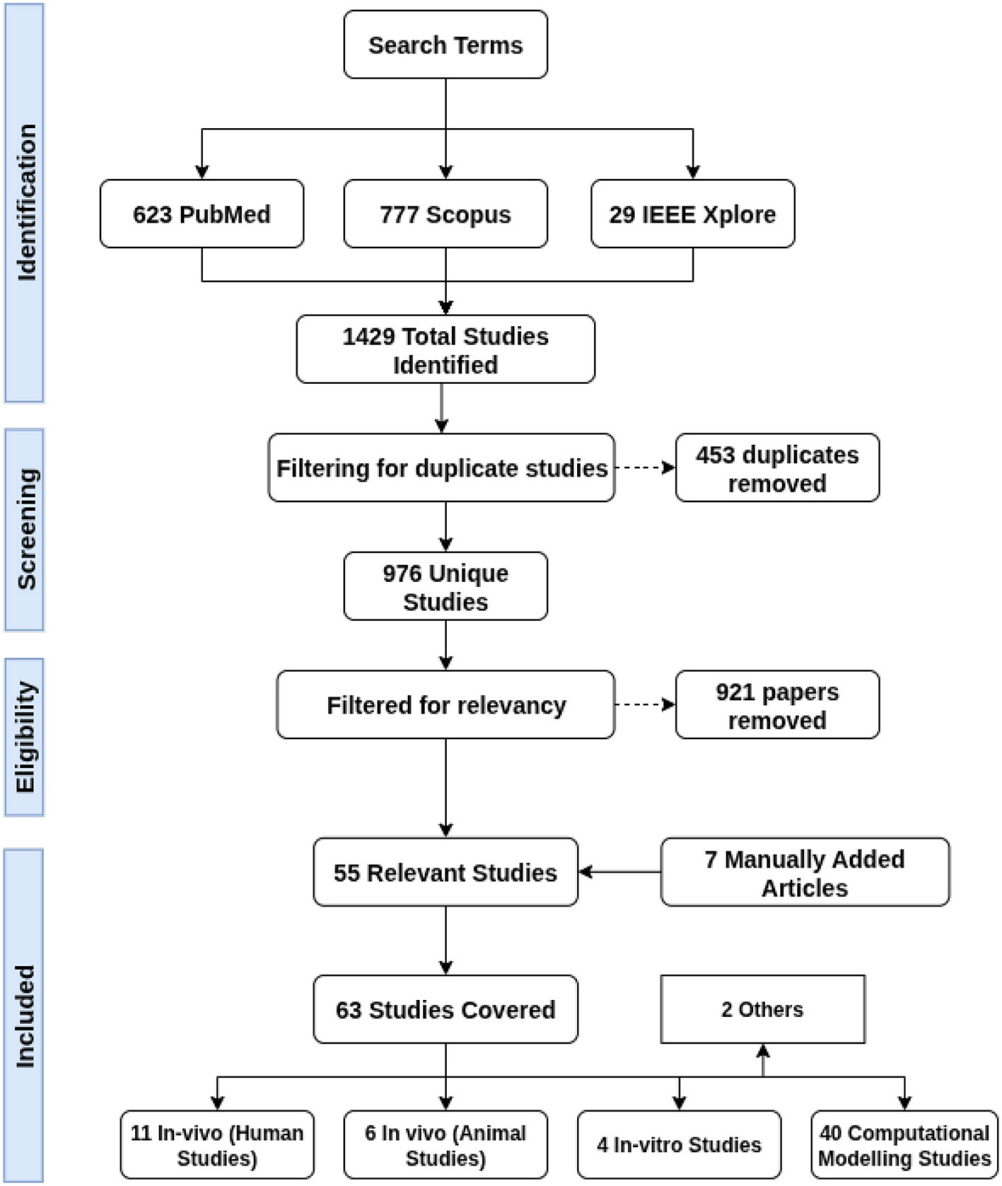
A flowchart detailing the process used for obtaining and filtering the papers covered in this literature review.

We defined our search phrase as: “‘Non-invasive deep brain stimulation' OR ‘temporal interference stimulation' OR ‘interferential stimulation' OR ‘interfering electric fields”' AND “brain.” Inclusion criteria were peer-reviewed journal articles employing TI for sub-threshold deep brain modulation, where studies investigating supra-threshold stimulation were excluded. Other exclusion criteria were non-brain applications, non-English publications, review articles, studies primarily focused on hardware development and non-electrode-based stimulation methods. We searched PubMed, IEEE Xplore, and Scopus for studies published between 2017 and 2024. This search yielded 1,429 studies. After removing duplicates and applying our inclusion criteria, 55 relevant studies were included, and an additional 6 studies were added manually. A detailed overview of our search and filtering process is shown in Figure 3.

**Figure 4:**
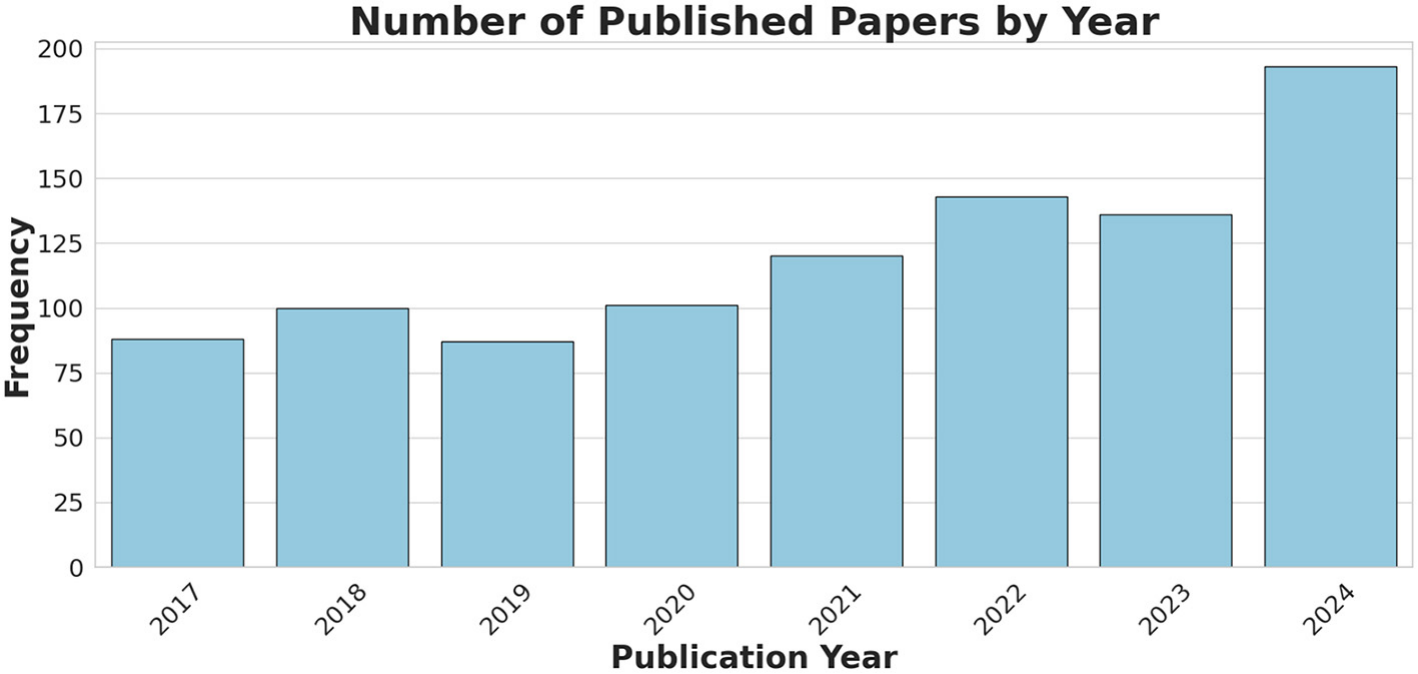
A bar chart showing the number of the published TI-related papers (after duplicate removal), obtained through the keyword search, covering the period from Jun 2017 to Dec 2024.

Our search results show that since its introduction in 2017, TI has gained increasing interest in the research community. Publications on TI have grown significantly, as illustrated in Figure 4, which displays the number of studies, after removing duplicates, published each year from 2017 to Dec 2024.

## 4 TI safety evaluation

The use of electric stimulation dates back to the Roman Empire (28, 29), with Luigi Galvani's 18th-century work on tDCS laying the groundwork for its clinical applications (30). Building on these foundations, this section reviews studies that focus on TI's tolerability and safety in humans. We have also included a summary of the in-vivo human TI studies, which can be found in Table 1. We will then discuss the current literature gaps on TI safety and what future research could follow to progress the field further.

**Table 1:** Summary of *in-vivo* human studies using temporal interference stimulation (TI).

**Study**	**Design**	**Population**	**Target**	**Control**	***Δf* (Hz)**	**Carriers (kHz)**	**Sessions**	**Primary outcome**	**Notes**
Zheng et al. (31)	RCT, double-blind	40 Healthy adults (male)	M1 leg area	Sham	20	2.0 & 2.02	10 sessions	Vertical jump height	↑ CMJ, ↑ SJ in TI vs sham
Wessel et al. (32)	Cross-over, RCT, Double-blind	45 Healthy adults and 15 Elderly	Striatum	HF & TMS control	100	2 & 2.1	Exp.1: 4 sessions; Exp. 2: 2 session; TMS Control: 1 session	Motor learning (SFTT)	TI increased motor-network activation and accelerated motor learning
Violante et al. (7)	Cross-over design, Double-blind	52 Healthy adults	Hippocampus	Sham	5	2.0 & 2.005	Exp.1: 1 session; Exp.2: 2 sessions	Episodic memory accuracy	TI enhanced episodic memory accuracy
Ma et al. (33)	Cross-over design, Single-blind	29 healthy adults	M1 hand area	Sham	20 & 70	2 & 2.02; 2 & 2.07	Three sessions	RT, MEP amp., learning	70Hz: ↑ RT & IO slope; 20Hz: ↑ learning & MEP amp.
Zhang et al. (34)	RCT, Single-blind	60 young adults	Right frontoparietal	TI-sham & tACS-sham	6	2.0 & 2.006	One session	N-back accuracy / RT / IES	TI was feasible, blinded, with WM gains comparable to tACS
Zhu et al. (35)	RCT, double-blind	40 young adults	M1	Active comparator (tDCS)	20	2 & 2.02	Two sessions	FC	Both TI and tDCS ↑ FC M1–premotor/SMA; no between-group difference
Von Conta et al. (36)	Randomized Cross-over, Single-blind	33 healthy adults	Parieto-occipital cortex	HF control (1kHz mono)	IAF	1	Three sessions	α-power after-effect	No significant difference between TI, tACS, and control
Iszak et al. (21)	Pseudo-randomized Crossover design	24 Healthy adults	Muscle / retina / occipital EEG	TACS, Carrier only, Sham	10 (TI)	2.000 & 2.010	Exp.1: 1 session; Exp.2: 1 session; Exp.3: 2 sessions	Muscle twitches, phosphenes, EEG α power	Demonstrated deeper focus without phosphenes in TI; EEG α-modulation observed only in tACS
Piao et al. (37)	Single-blind, RCT	38 healthy adults	Left M1	Sham (no current)	20 or 70	2 & 2.02; 2 & 2.07	Three sessions (single visit)	EEG, NSE, batteries, AEs	Safe, no NSE change; stable EEG
Vassiliadis et al. (38)	RCT, Double-blind	119 young / older adults and TBI patients	Striatum & hippocampus	Placebo (HF)	100	2 & 2.1	1–4 sessions/Exp.	Safety and blinding feasibility	TI was safe, well-tolerated and effectively blinded

This table presents key characteristics of published studies that have applied temporal interference stimulation (TI) in human participants. It includes information on study design, population, stimulation targets, sample sizes for TI and control conditions, control types, stimulation parameters (including envelope frequency Δ*f*, carrier frequencies, and current per electrode pair), session durations, primary outcome measures, reported effect metrics, and results. TI, Temporal Interference; tDCS, transcranial Direct Current Stimulation; tACS, transcranial Alternating Current Stimulation; HF, High Frequency; M1, Primary Motor Cortex; SMA, Supplementary Motor Area; fMRI, functional Magnetic Resonance Imaging; RT, Reaction Time; MEP, Motor Evoked Potential; FC, Functional Connectivity; CMJ, Countermovement Jump; SJ, Squat Jump; IES, Inverse Efficiency Score; AEs, Adverse Event; TBI, Traumatic Brain Injury; Exp., Experiement; IAF, Individual Alpha Frequency; NSE, Neurological Safety Biomarkers.

### 4.1 Safety profiles and adverse events

While some studies have reported functional outcomes of TI in human cohorts (31–34, 38–40), only a limited subset of studies have directly assessed its safety and tolerability across varying stimulation parameters (10, 37, 41). Until now, 412 participants have undergone TI stimulation with a current strength ranging from 0.5–4mA and stimulation beat frequencies ranging from 20 to 130 Hz, with a carrier frequency of 2 kHz and a stimulation duration of up to 40 min. Collectively, the studies evaluated the safety and tolerability of different types of TI protocols, which are primarily classified as either constant or burst. Constant stimulation protocols deliver a continuous electrical current, leading to sustained neural activation. In contrast, burst stimulation protocols apply intermittent bursts at a predefined frequency (32).

No serious adverse effects were reported across any of these studies for younger and older adults. Similar to healthy populations, clinical populations such as patients with traumatic brain injury also did not experience any serious adverse effects, with only 1 in 15 patients dropping out of the study due to experiencing strong sensations reminding the patient of their traumatic brain injury (41). Reports of transient sensory experiences, including tingling, fatigue, itching, vibration, or pressure, were confined to the highest current dose (2 mA). These sensations were consistent between the active and placebo TI conditions and are also in line with the sensations previously associated with traditional tES techniques, such as tDCS and tACS (42–47). Impedance and temperature measurements confirmed safe skin-electrode interactions (37).

Blinding effectiveness is essential for TI stimulation's clinical use. Vassiliadis et al. reported that participant sensations between active and placebo sessions were similarly rated in intensity at 2 mA, with guesses about session order no better than random, demonstrating potential blinding efficiency (41). Conta et al. also confirmed TI's tolerability and blinding effectiveness at 1 mA, underscoring the value of maintaining robust placebo controls for diverse applications (48).

### 4.2 Limitations and future research directions for safety evaluation of TI

While existing studies have reported no significant short-term adverse effects, the long-term safety of TI stimulation remains largely unexplored, necessitating further investigation through more inclusive and longitudinal research designs. Factors such as age, psychological conditions, and pre-existing health issues can significantly influence how TI-generated electric fields propagate through the brain and other tissues, highlighting the importance of studying diverse populations (20). Moreover, as Cassara et al. have noted, there is a critical discrepancy between the theoretically safe current amplitudes and those commonly employed in experimental settings, underscoring an urgent need for standardized, TI-specific safety guidelines (49). To ensure these guidelines are robust and relevant, they should be grounded in physiological metrics, such as skin sensation thresholds and tissue heating effects, rather than being adapted from frameworks designed for tACS and tDCS, which may not fully capture the unique characteristics of TI stimulation.

Addressing these safety concerns is vital not only to minimize potential risks associated with long-term use but also to improve the reliability and reproducibility of TI studies. Establishing comprehensive safety standards tailored to TI will enable researchers to optimize stimulation parameters, ensure consistent application across studies, and unlock the full potential of this promising technology for therapeutic and research purposes.

## 5 Comparative analysis of temporal interference

This section analyses studies that investigated the neuronal responses generated by TI stimulation, highlighting the critical parameters that influence these responses. Subsequently, studies comparing the efficacy of TI to established transcranial neuromodulation methods—including tACS and tDCS—are reviewed.

### 5.1 Investigating neuronal responses to TI and key influencing parameters

Building on the original experiment described by Grossman et al. (4), several research groups have conducted *in-vivo* rat experiments and computational analyses to enhance our understanding of TI's impact (12, 13, 50–53). A multi-scale computational model by Gomez et al. (50) explored the effects of TI stimulation across various biological scales, from molecular interactions to whole-organism responses. The finite-element-based computational study showed that TI stimulation could theoretically and selectively target deep brain structures without affecting superficial cortical areas. The simulations also validated that the effectiveness and location of stimulation can be adjusted by modifying the electric currents' carrier frequency and amplitude ratio between the electrode pairs, which is consistent with previous literature (4). A detailed summary of the conducted experiments and their results can be found in Table 2.

**Table 2:** Summary of results from the computational multi-scale mouse model study by Gomez et al. (50).

**Experiment**	**Conditions**	**Results**
Beat frequency effect	Beat frequencies: 5–100 Hz, constant carrier frequency: 2 kHz	No influence on activation threshold within the tested range
Carrier frequency effect	Carrier frequencies: 1–4 kHz, constant beat frequency: 10 Hz	Activation threshold increased with higher carrier frequencies
Amplitude ratio effect	Amplitude ratios: various (e.g., 1:1, 2:1), constant carrier and beat frequencies	Location of nerve activation shifted according to amplitude ratio

The experiments varied beat frequencies, carrier frequencies, and amplitude ratios of TI to assess their impact on neural activation. Beat frequency did not influence the activation threshold, while higher carrier frequencies increased it. The location of nerve activation changed with different amplitude ratios.

Modak et al. evaluated non-invasive TI stimulation using two 2 and 2.02 kHz currents with a 20 Hz beat frequency aimed at the left caudate in healthy adults during resting-state fMRI, but instead observed significant BOLD increases in bilateral orbitofrontal cortex and fusiform/parahippocampal regions, with modeling showing peak fields overlapping the orbitofrontal cortex rather than the intended target (54). They also noted deactivation in the right precuneus/superior parietal lobule, suggesting off-target conduction effects. Even so, they found no significant differences in discomfort between active and sham conditions. Only one participant reported a mild headache lasting up to 15 days, indicating TI is generally safe and well tolerated (54).

A number of *in-vivo* and *in-silico* experiments were conducted to investigate the mechanism of TI and its key influencing parameters (7, 13, 51, 52). Two *in-vivo* studies involving rats and humans revealed that in both populations, neuronal firing rates exhibited increased sensitivity to unbalanced current ratios (7, 51). Specifically, this sensitivity was significant in rats at carrier frequencies below 1,800 Hz (51). Violante et al. investigated the impact of different current ratios on Blood Oxygenation Level Dependent (BOLD) signal and memory performance. Their results indicate that a 1:3 current ratio significantly reduced the BOLD signal and improved episodic memory, as measured by a face-name paired associative task (7). Further *in-silico* experimentation showed that deeper targets could benefit from lower carrier frequencies due to their effects on the neuronal activation threshold and sensitivity to amplitude modulation (13, 52). Across all human studies discussed in this section, TI stimulation was well tolerated, with no serious adverse effects reported. Only a few mild and common side effects were noted by Violante et al. and Modak et al. (7, 13, 51, 52, 54). In a crossover behavioral study comparing active TI with sham sessions, the only side effect occurring significantly more often during TI was itchiness at the electrode site (*Z* = −2.354, *P* = 0.019) (7).

#### 5.1.1 Limitations of cross-species comparison

While both human and rodent studies provide valuable insights into TI-evoked neural effects, direct comparison is constrained by key interspecific differences. Rodent skull thickness, tissue conductivities, and brain geometry differ substantially from humans, altering the electric field distribution and required current densities (55). Moreover, anesthesia protocols, used in animal studies, can modify neuronal excitability and vascular dynamics, which may not translate to awake human participants (56). Lastly, behavioral and electrophysiological outcome measures—such as motor evoked potentials in rats versus cognitive or perceptual readouts in humans—use different metrics and scales, limiting direct alignment of safety and efficacy thresholds. Together, these factors necessitate caution when extrapolating parameter settings and effect sizes from rodent models to clinical TI applications.

### 5.2 Evaluating the potential of TI stimulation compared to traditional tES techniques

Recent studies have evaluated the effectiveness of TI stimulation compared to established non-invasive brain stimulation approaches, such as tDCS and tACS (18, 35, 53, 57–59). Early findings are promising, suggesting that TI has the potential to target deep-brain regions with minimal impact on the surrounding cortical areas. In particular, three recent *in-vivo* studies targeting the primary motor cortex (M1) in humans and rats reported that TI significantly enhances motor skills and outperforms tACS and tDCS in improving motor performance (31, 35, 58). However, these results are not entirely consistent. While some studies indicate that TI offers superior enhancements in functional connectivity and motor skills compared to traditional tDCS (35, 58), others report only marginal improvements (57).

Current research primarily focuses on single-session, short-term effects, leaving the long-term outcomes of TI stimulation largely unexplored. Moreover, many findings rely on computational models, which can introduce inconsistencies due to simplifications, such as omitting cerebrospinal fluid to accommodate hardware limitations (57).

Although the mean e-field strength of TI is comparable to other tES methods, achieving sufficient depth often requires higher intensities, potentially exceeding comfort thresholds and increasing the risk of conduction block (19, 20, 53). A recent study comparing advanced techniques such as HD-tACS and Intersectional tACS highlighted their potential to stimulate deep brain regions (57). However, the authors noted that with further optimisation, TI could achieve greater efficacy by better confining the stimulation field to the target area (57). This potential for further improvement of TI was supported by one more study, comparing tACS to TI (18). Conversely, early computational studies pointed to higher off-target effects in TI, particularly in deep brain stimulation applications, emphasizing the need for improved focality to enhance its clinical utility (60).

As highlighted by Conta et al. (20), variability in the generated e-field distribution presents another challenge for TI stimulation, resulting in inconsistent stimulation effects across individuals. Despite emphasizing the potential benefits that TI can offer, the team underscores the need for individualized stimulation montages in order to better leverage the technology to its full potential (20). Similarly, Zhu et al. reported insufficient stimulation intensity and a limited range of experimental conditions, restricting the exploration of TI's full potential (35). Furthermore, Qi et al. discussed the translational challenges of applying results from animal models to humans, noting the differences in brain complexity and size that complicate achieving precise and effective deep brain stimulation in clinical settings (58).

## 6 Optimisation of TI parameters

### 6.1 Electrode configurations and objective functions for enhanced precision

The development of TI stimulation relies heavily on the optimisation of key parameters to achieve precise and effective neuromodulation. While demonstrating the advantage of electrode arrays over conventional electrodes in generating more localized stimulation hotspots, Huang's study highlighted the necessity of numerical optimisation in TI due to the complex relationship between electrode placement and the formation of the resulting e-field distribution (61). To visualize the dynamic nature of TI, Huang deviated from traditional static visualization techniques and adopted a dynamic approach to his simulations, tracking the evolution of the e-field distribution over time, rather than focusing on a single snapshot in time (61). Building on the need for precise adjustments in TI parameters—such as electrode placement, current intensity, phase, and frequency—Lee et al. conducted an exhaustive search across three realistic head models to optimize the “Peak Ratio,” defined as the ratio of e-field amplitude between the target region and cortical areas (62). By evaluating configurations across 61 possible electrode pairs and current levels, they identified setups that maximized the Peak Ratio, thereby improving stimulation precision in the desired brain regions.

Huang et al. extended this framework by introducing a constraint within their optimisation function to minimize off-target e-field effects (63). This added constraint enhanced the precision of TI by focusing on both maximizing stimulation in target areas and minimizing unintended effects in non-target regions (63). Expanding further, Lee et al. introduced the “Peak-Sum Ratio” objective function, defined as the ratio between the maximum e-field amplitude at the target region and the mean amplitude in neocortical regions where the amplitude exceeds 90% of the peak amplitude. This refined objective function, coupled with the sequential addition of electrode pairs, enabled targeted focality enhancements in regions like the hippocampus across three realistic head models (22).

These studies collectively underscore the critical role of defining tailored optimisation metrics, called objective functions, in achieving optimal TI stimulation. Such precision is essential for improving efficacy in targeted brain areas and reducing unintended stimulation in other areas, thus supporting the development of safer and more effective non-invasive neuromodulation techniques.

### 6.2 Advances in electrode configuration and neural response tailoring

To advance TI parameter optimisation, recent strategies include Cao and Grover's Hodgkin-Huxley model (64), which uses electrode pairs to improve spatial precision, and Missey et al.'s orientation-tunable approach (65) that adjusts stimulation thresholds based on axonal alignment and neuron types.

Cao and Grover's model employs multiple electrode configurations to create dynamic, steerable stimulation patterns, enhancing focality and minimizing off-target effects (64). Their approach introduces “patch-pairs” of electrodes that focus currents into targeted brain areas while reducing unintended stimulation, with findings indicating that different neuron types, such as parvalbumin-expressing inhibitory neurons, may require tailored stimulation strategies (64). Building on these insights, Missey et al. demonstrated that axonal alignment relative to the electric field significantly affects stimulation thresholds, with parallel axons requiring lower current for activation compared to perpendicular ones, highlighting the importance of electrode orientation for precise, minimally invasive deep brain stimulation (65).

### 6.3 Evolutionary algorithms for TI parameter optimisation

Several studies have employed genetic algorithms (GAs) to address the complexities in optimizing TI parameters. Honarbakhsh and Mohammadzadeh developed a GA-based method to increase spatial resolution by defining an objective function that minimizes off-target e-field exposure while significantly expanding the number of electrode sources, thereby enhancing focal precision in TI applications (66). Similarly, Stoupis and Samaras used GA-based methods to optimize the electric field ratio between the target region's gray matter and surrounding brain tissue (67). These studies show that genetic algorithms are potentially valuable for managing high-dimensional, complex, multi-objective, and non-convex TI optimisation problems.

Building on this, Wang et al. proposed multi-objective optimisation via the evolutionary algorithm (MOVEA) framework, which balances focality and intensity by generating a Pareto front of optimized solutions. This flexible approach explores electrode configurations without predefined constraints, highlighting the potential of evolutionary algorithms in optimizing complex, multi-objective TI scenarios where trade-offs must be carefully managed (68).

### 6.4 Artificial neural networks for TI parameter optimization

With the recent popularity of deep learning and Artificial Neural Networks (ANN) (69), Karimi et al. extended the computational modeling approach using an ANN framework to estimate TI parameters in two simplified head models: homogeneous and inhomogeneous cylinders (70). The inhomogeneous model incorporated layers representing the scalp, skull, cerebrospinal fluid, and brain tissue. The researchers explored two electrode configurations: one with two electrode pairs and another with four electrode pairs. To train the ANN, they generated two datasets by pre-calculating e-field distributions based on systematically varied stimulation parameters. For the two-electrode configuration, a dataset of 474 samples was prepared, with the network inputs representing the coordinates of the activated area's center of gravity, and the outputs including the total current and current ratios between electrode pairs. For the four-electrode pair configuration, a larger dataset of 15,755 samples was developed, incorporating similar parameter variations while keeping the current ratio fixed for two of the electrode pairs. The ANN demonstrated successful control over the position and shape of the stimulated areas by adjusting the TI stimulation parameters, showcasing its potential for precise neuromodulation (70).

Building on artificial intelligence-based optimisation, Bahn et al. introduced unsupervised neural networks (USNNs) to refine the currents of high-definition electrodes, targeting focal stimulation in deep brain regions (71). Their approach involved a two-part neural network architecture: the first component generated electrode currents, while the second estimated interference exposure based on those currents. These two components operated in tandem, with the exposure network assessing how well the stimulus aligned with the target area and using this feedback to iteratively adjust the weights of the current-generation network through backpropagation. The authors argued that the proposed USNNs offered superior nonlinear optimisation capabilities, enabling to navigate the intricate relationships between electrode placement and stimulation patterns more effectively than traditional methods. Additionally, the flexible network architecture and a targeted loss function—designed to prioritize peak accuracy, concentration, and minimization of off-target effects—were other potential key advantages of the proposed solution (71).

### 6.5 Challenges and future directions in TI optimisation

Computationally intensive optimisation methods, such as exhaustive search and evolutionary algorithms, are promising but often time-consuming and may not always provide globally optimal solutions (62, 66–68). The need for multiple electrodes to achieve optimal focality is also limited by the channel capacities of current stimulation devices (64). While incorporating neuronal fiber orientation to optimize the e-field has proven effective, this requires magnetic resonance imaging data, which is often inaccessible and time-consuming to obtain (65). Additionally, deep learning methods, though efficient when successful, lack transparency, especially when training datasets and models are not publicly available. The heavy reliance on simulations without sufficient experimental validation raises concerns about the real-world applicability of these findings.

Future research should focus on more efficient optimisation algorithms that balance computational demands with solution quality, potentially through hybrid approaches. Moreover, current methods often overlook the impact of carrier frequency on neuronal excitability, which has been shown to be a key factor (13, 50). Importantly, there is a pressing need for open-source sharing of training datasets, Finite Element Method (FEM) models, and optimisation models in TI for further validation and replicability.

Current objective functions used in TI optimisation often focus on a narrow set of parameters, such as focality and intensity, without fully accounting for the dynamic nature of neuronal excitability or individual variability. While addressing these limitations, it is crucial to delineate the roles of different components in the optimisation process. Factors such as neuron-specific responses, regional differences in tissue properties, and the effects of carrier frequency modulation are better incorporated at the e-field modeling stage, where a more accurate and biologically informed representation of neuronal behavior can be achieved. Objective functions, in turn, should focus on leveraging these improved models to target clinically relevant outcomes, such as maximizing therapeutic efficacy while minimizing side effects. Additionally, hybrid optimisation methods integrating experimental validation and real-world data could help refine these objective functions for better clinical applicability. Furthermore, there is a need for optimisation studies to use real and more detailed, MRI-derived brain images, instead of synthetic or averaged models, which is evident by looking at Table 3, as well as supported by the study of Wang et al., which is underlying the fact, that more detailed models will lead to significantly different results (72).

**Table 3:** Head Model Types used in the reviewed optimisation papers.

**References**	**Head Model Type**
Lee et al. (62)	MRI-derived head models from 3 healthy male participants.
Huang, (61)	Standard MNI-152 average head.
Missey et al. (65)	No human head model (mouse hippocampus network).
Bahn et al. (71)	Population Head Models repository: 38 MRI-derived head meshes.
Huang et al. (63)	ICBM-152 v6 template head.
Karimi et al. (70)	Analytical homogeneous vs. inhomogeneous synthetic models.
Esmaeilpour et al. (13)	The origin of the head model was not stated.
Stoupis and Samaras, (67)	Population Head Models repository: 38 realistic head meshes.
Wang et al. (19)	Individual MRI derived FEM models (subject specific segmentation).
Wang et al. (72)	Both synthetic spherical and MRI derived models.

## 7 New TI modalities

Although TI is relatively new, several innovative modalities are continuously emerging, enhancing the original interferential method proposed by Grossman et al. (4). This section briefly discusses how some of these advancements are pushing the boundaries of non-invasive brain stimulation. A summary of the studies covered in this section can be found in Table 4.

**Table 4:** Comparison of novel TI modalities: key metrics and trade-offs.

**Modality**	**Envelope control**	**Focality change vs. TI**	**Max e-field (V/m)**	**Current limitation**
Standard TI	Continuous beat	Baseline	0.5	N/A
PMI (16)	Pulse-like	+0%	0.5	Phantom only
Epicranial TI (73)	Continuous	+9%	1.9	Invasive
Gigahertz TI (74)	Continuous	NS	Up to 12	Invasive
Epidural TI (75)	Continuous	NS	1	untested *in vivo*
PWM-TI (76)	Duty-cycle	±0%	0.5	Modeling only
STFS (77)	Arbitrary	NS	NS	untested *in vivo*
MTI (78)	Continuous	Steerable	NS	Modeling only

TI, Temporal Interference; PMI, Phase Modulation Interference; E-field, Electric Field; STFS, Spatio-Temporal Fourier Synthesis; MTI, Multi-Point Temporal Interference; PWM-TI, Pulse-Width Modulated Temporal Interference; NS, Not Stated.

### 7.1 Phase modulated temporal interference

Terasawa et al. introduced Phase Modulation Interference (PMI), allowing transient changes in the stimulation envelope's amplitude, achieving finely controlled, pulse-like stimulation envelopes (16). These results were obtained through a simulation study and later confirmed by a tissue phantom experiment, where both the simulations and experimental measurements showed consistent spatial distributions of the envelope modulation amplitude, validating the computational model with real-world experimental data.

### 7.2 Minimally invasive temporal interference

Invasive TI approaches, such as epicranial cortical stimulation, can improve e-field targeting, enhancing its intensity by up to 3.8 times and increasing focality by 9% compared to transcranial TI methods (73, 79). Additionally, deep brain stimulation using temporally interfering electromagnetic waves in the gigahertz range, generated by endocranially implanted antenna arrays, can bypass scalp attenuation, achieving higher focality and e-field intensities at deep brain targets, up to 12 V/m (74).

Lee et al. evaluated the feasibility of minimally invasive epidural temporal interference (eTI) for deep brain stimulation using a combination of computational simulations and phantom model experiments (75). By modeling e-fields, they optimized eTI to selectively target the Anterior Hippocampus, Subthalamic Nucleus, and Ventral Intermediate Nucleus of the Thalamus. Their simulations suggested that an e-field amplitude of 1 V/m could be achieved with a 5.6 mA current, within safety ranges from prior epidural studies in stroke and depression (80, 81). Validation through a skull phantom, filled with brain-mimicking agarose gel and outfitted with copper electrodes, showed close agreement between simulated and experimental e-field distributions. Although promising, further testing, including temperature and *in-vivo* studies, is required to assess clinical safety and tolerability fully.

### 7.3 Pulse-width modulation

Luff et al. developed Pulse-width Modulated Temporal Interference (PWM-TI), a non-invasive technique that modifies electrical field parameters through pulse-width modulation rather than traditional sinusoidal signals (76). Using *ex-vivo* and *in-vivo* methods, including computational modeling and mouse studies, they showed that PWM-TI performs similarly to conventional TI. Their findings indicate that the interaction between pulse-width modulation and cell membrane time constants influences the resulting electric field dynamics. This suggests that varying the duty cycles of the signal could enable targeted modulation of specific cell types.

### 7.4 Spatio-temporal Fourier synthesis

Beyond conventional temporal interference approaches, recent advancements have explored novel stimulation paradigms leveraging the principles of spatio-temporal Fourier synthesis (STFS) (77). Kish et al. introduced a method that utilizes multiple electrode pairs driven by harmonically structured sinusoidal currents to enhance both the spatial and temporal precision of deep-brain stimulation (77). Unlike traditional TI, which relies on high-frequency beating sinusoidal currents, STFS constructs sharp, localized stimulation spikes while maintaining low stimulation intensity at the scalp (4, 77). Computational simulations have demonstrated that STFS can generate various signal configurations, including high-frequency prime harmonics, quasi-random noise, and chirped waveforms, each offering unique stimulation profiles. While this technique shows promise for achieving targeted neuromodulation with improved focality, further validation in experimental and clinical settings is necessary to refine stimulation waveforms and assess safety and efficacy.

### 7.5 Multi-point temporal interference stimulation

Multi-Point Temporal Interference (MTI) stimulation builds upon traditional TI by introducing a method to simultaneously and independently target multiple deep brain regions (78). By assigning distinct frequencies to each electrode, MTI avoids the need for additional electrode pairs, streamlining its application for both research and clinical settings. This modality optimizes stimulation parameters, including frequency and amplitude, to minimize artifacts while ensuring precise e-field strength across multiple targets. Validated through several computational models of varying complexity as well as tissue phantom experiments, MTI demonstrated the ability to generate independent, steerable stimulation points. This advancement offers a potentially versatile tool for modulating complex brain networks involving deep structures, expanding the capabilities of non-invasive neuromodulation (78).

### 7.6 Challenges and future perspectives in new TI modalities

Despite the promising advancements in new TI modalities, several challenges remain in fully realizing their potential for non-invasive brain stimulation. Precise targeting of deep brain regions while minimizing effects on surrounding tissues is a key hurdle. Recent studies have highlighted that inter-individual anatomical variability can shift the focal spot of stimulation significantly, making fixed montages unreliable without per subject optimisation. However, purely individualized optimisation demands high resolution MRI and heavy computation, whereas population based templates can match personalized optima within 1~7%, offering a more scalable compromise (82, 83). Techniques like single pulse TI and pulse width modulated TI offer improved control over stimulation parameters, but optimizing spatial resolution remains an active area of research (76, 82–84). Safety concerns, particularly regarding the long-term effects of high-frequency stimulation, require thorough clinical trials to assess potential risks and side effects (73, 79). The reliance on computational models to optimize electrode configurations demands empirical validation to ensure their accuracy in real-world applications (74, 78). A deeper understanding of the underlying neural mechanisms is critical for tailoring interventions to specific neurological conditions. Addressing these challenges through targeted research is key to translating new TI modalities into clinical practice.

## 8 Clinical applications of TI assessed in humans

Although TI has extensive potential applications, only limited studies have evaluated its efficacy in humans, focusing on motor learning, working memory, epilepsy, and Parkinson's Disease (7, 31, 32, 38, 85, 86).

Two complementary studies investigated TI's effect on motor learning via targeting the striatum. Both found that TI successfully modulates motor performance. Wessel et al. observed that theta-burst stimulation using burst protocol improved motor performance and striatal-frontal connectivity in 15 young, healthy individuals, with similar results in a cohort of older adults (32). Similarly, Vassiliadis et al. showed that 80 Hz TI enhanced motor skill acquisition and connectivity in 48 healthy volunteers, assessed through a force-tracking motor learning task (38). Building on these findings, Zheng et al. focused on the Primary Motor Cortex (M1) and demonstrated that TI stimulation effectively enhances motor excitability and performance in humans (31). Using both computational and experimental approaches (*N* = 40), they showed significant improvements in vertical jump performance, including countermovement and squat jump heights.

In a sham-controlled trial, Zhang et al. assessed TI's impact on working memory compared to tACS and sham (34). While TI showed effective blinding, it resulted in only slight working memory improvements under high cognitive load, with no significant differences between TI and other stimulation methods. The study, though confirmed TI's safety and potential, indicated the need for further research with larger sample sizes.

Finally, Acerbo et al. explored TI as a non-invasive alternative to deep brain stimulation for epilepsy, demonstrating its ability to target deep brain structures, such as the hippocampus. In a cadaver-based study, TI at an envelope frequency of 130 Hz reduced pathological biomarkers, including a more than 50% reduction in fast ripples, suggesting its potential for treating epilepsy (85).

### 8.1 Limitations and future directions

The first-in-human applications of TI have shown promising potential in areas such as motor learning and epilepsy. However, several limitations must be addressed to fully harness its clinical potential. Importantly, most TI studies lack a clear understanding of the neural mechanisms underlying TI's effects. Future research should aim to identify the specific brain regions and networks targeted by TI and how these changes correlate with behavioral outcomes. Techniques such as functional magnetic resonance imaging or electroencephalography could be used to explore these real-time brain changes, with some pilot studies already being conducted (7). Addressing these limitations and expanding our understanding of TI effects will be key to realizing its full potential.

Another limitation is the sample size and diversity of the existing studies, which predominantly involve healthy young adults. To enhance the applicability of TI, future research should include larger, more diverse populations, including individuals with neurological conditions. The focus on short-term effects is another limitation. While initial results are promising, little is known about the long-term safety and efficacy of TI. Therefore, future research should expand participant demographics to include a diverse range of ages, health status, and racial backgrounds, enhancing the generalisability of the findings. Longitudinal studies are needed to assess the durability of TI's effects and identify any potential risks associated with prolonged use.

## 9 Conclusion

This review highlights TI's potential in targeting deeper brain structures not traditionally reachable by tES, indicating a potential to serve as an alternative treatment for neurological conditions. Nevertheless, significant challenges remain that must be overcome to advance the technology's development. Clarifying the definition of TI-sham and understanding the effects of high-frequency sham on the brain is vital for accurate control conditions. Additionally, identifying the optimal envelope frequency and determining the maximum safe injection current is crucial for maximizing efficacy and safety.

The current research on TI stimulation faces several limitations that need to be addressed to enhance its accuracy, reproducibility, and translational potential. One major challenge lies in the reliance on computational models, which, while beneficial, often involve approximations that can skew results. Models lacking sufficient anatomical detail, such as the omission of cerebrospinal fluid or variations in tissue conductivity, can lead to inaccuracies in predicting e-field distributions and stimulation outcomes. To mitigate this, future models should incorporate more detailed anatomical and biophysical features, improving the fidelity of simulations and their alignment with empirical results (72). Additionally, anatomical variability between individuals presents a challenge to achieving consistent and comparable results. TI studies have yet to fully account for these variations, which can significantly influence the e-field distributions and, thus, stimulation outcomes. Future research should focus on addressing individual differences between patients to ensure that the same brain regions are stimulated. Furthermore, this personalisation needs to take into account the effects of the stimulation area and its effects on the e-field intensity.

Finally, there is a significant gap in understanding TI's long-term effects. Currently, studies focus on short-term stimulation effects, leaving potential long-term impacts largely unexplored. Expanding the duration of these studies could offer crucial insights into TI's performance over extended periods. This duration increase could be in the form of repeated visits, exploring the longevity of TI-induced effects, or—focusing on longer protocols, exploring the effects of e-filed saturation in the brain and its associated physiological and behavioral changes. In addition, there is a pressing need to increase sample sizes in human and animal studies. Larger sample sizes would allow for a more robust examination of how TI's parameters vary across different individuals, thus providing a clearer understanding of its effects. Addressing these issues systematically will be crucial in leveraging TI's full potential as an effective neuromodulation method.

Future research must continue to develop personalized protocols, leveraging computational models to refine stimulation parameters. Extensive clinical trials are essential to validate TI's efficacy across diverse populations. While TI represents a frontier in non-invasive brain modulation, its advancement depends on a deeper understanding of its mechanisms, technological innovation, and rigorous clinical validation.

Despite the extensive research on optimisation and personalisation, a critical knowledge gap persists: how exactly do techniques like TI stimulation interact with brain tissue and influence the complex networks within the brain? Even with tDCS—a technology that has been in use for a while—our understanding of its precise mechanisms of action is still incomplete. This underscores an urgent need for more comprehensive, multidisciplinary research. Clinicians, biophysicists, neurologists, neuroscientists, and engineers must come together to unravel these intricacies. By integrating insights from various fields, we can paint a clearer picture of how TI and other brain stimulation techniques affect different regions, cell types, and the emergent properties of neural networks. Understanding the working principles of TI—much like any form of neuromodulation—requires bridging the knowledge from different domains to unlock its full potential.

## References

[1] Gomez-Tames J Fernández-Corazza M. Perspectives on optimized transcranial electrical stimulation based on spatial electric field modeling in humans. J Clin Med. (2024) 13:3084. 10.3390/jcm13113084 38892794 PMC11172989

[2] Datta A Bansal V Diaz J Patel J Reato D Bikson M. Gyri-precise head model of transcranial direct current stimulation: improved spatial focality using a ring electrode versus conventional rectangular pad. Brain Stimul. (2009) 2:201–7. 10.1016/j.brs.2009.03.005 20648973 PMC2790295

[3] Thair H Holloway AL Newport R Smith AD. Transcranial direct current stimulation (tDCS): a beginner's guide for design and implementation. Front Neurosci. (2017) 11:641. 10.3389/fnins.2017.00641 29213226 PMC5702643

[4] Grossman N Bono D Dedic N Kodandaramaiah SB Rudenko A Suk HJ. Noninvasive deep brain stimulation via temporally interfering electric fields. Cell. (2017) 169:1029–41. 10.1016/j.cell.2017.05.024 28575667 PMC5520675

[5] Hutcheon B Yarom Y. Resonance, oscillation and the intrinsic frequency preferences of neurons. Trends Neurosci. (2000) 23:216–22. 10.1016/S0166-2236(00)01547-2 10782127

[6] Mirzakhalili E Barra B Capogrosso M Lempka SF. Biophysics of temporal interference stimulation. Cell Systems. (2020) 11:557–72. 10.1016/j.cels.2020.10.004 33157010

[7] Violante IR Alania K. Cassarà AM, Neufeld E, Acerbo E, Carron R, et al. Non-invasive temporal interference electrical stimulation of the human hippocampus. Nat Neurosci. (2023) 26:1994–2004. 10.1038/s41593-023-01456-8 37857775 PMC10620081

[8] Currie A Wong J Okun M A. review of temporal interference, nanoparticles, ultrasound, gene therapy, and designer receptors for Parkinson disease. NPJ Parkinson's Dis. (2024) 10:195. 10.1038/s41531-024-00804-0 39443513 PMC11500395

[9] Fani N Treadway MT. Potential Applications of Temporal Interference Deep Brain Stimulation for the Treatment of Transdiagnostic Conditions in Psychiatry. Cham: Springer International Publishing. (2024). 10.1038/s41386-023-01682-5 PMC10700552 37524751

[10] Thiele C Tamm C Ruhnau P Zaehle T. Perceptibility and pain thresholds in low-and high-frequency alternating current stimulation: implications for tACS and tTIS. J Cogn Enhanc. (2025) 9:79–91. 10.1007/s41465-024-00304-2

[11] Plovie T Schoeters R Tarnaud T Joseph W Tanghe E. Nonlinearities and timescales in neural models of temporal interference stimulation. Bioelectromagnetics. (2025) 46:e22522. 10.1002/bem.22522 39183685

[12] Caldas-Martinez S Goswami C Forssell M Cao J Barth AL Grover P. Cell-specific effects of temporal interference stimulation on cortical function. Commun Biol. (2024) 7:1076. 10.1038/s42003-024-06728-y 39223260 PMC11369164

[13] Esmaeilpour Z Kronberg G Reato D Parra LC Bikson M. Temporal interference stimulation targets deep brain regions by modulating neural oscillations. Brain Stimul. (2021) 14:55–65. 10.1016/j.brs.2020.11.007 33186778 PMC9382891

[14] Thiele C Rufener KS Repplinger S Zaehle T Ruhnau P. Transcranial temporal interference stimulation (tTIS) influences event-related alpha activity during mental rotation. Psychophysiology. (2024) 61:e14651. 10.1111/psyp.14651 38997805

[15] Wang B Aberra AS Grill WM Peterchev AV. Responses of model cortical neurons to temporal interference stimulation and related transcranial alternating current stimulation modalities. J Neural Eng. (2023) 19:066047. 10.1088/1741-2552/acab30 36594634 PMC9942661

[16] Terasawa Y Tashiro H Ueno T Ohta J. Precise temporal control of interferential neural stimulation via phase modulation. IEEE Trans Biomed Eng. (2021) 69:220–8. 10.1109/TBME.2021.3091689 34161235

[17] Rampersad S Roig-Solvas B Yarossi M Kulkarni PP Santarnecchi E Dorval AD. Prospects for transcranial temporal interference stimulation in humans: a computational study. Neuroimage. (2019) 202:116124. 10.1016/j.neuroimage.2019.116124 31473351 PMC6819277

[18] Hirata A Akazawa Y Kodera S Otsuru N Laakso I. Electric field envelope focality in superficial brain areas with linear alignment montage in temporal interference stimulation. Comput Biol Med. (2024) 178:108697. 10.1016/j.compbiomed.2024.108697 38850958

[19] Wang G Dokos S. Selective myelinated nerve fiber stimulation via temporal interfering electric fields. In: 2021 43rd Annual International Conference of the IEEE Engineering in Medicine & *Biology Society (EMBC)*. IEEE (2021). p. 6033–6036. 10.1109/EMBC46164.2021.9630073 34892492

[20] von Conta J Kasten FH Ćurčić-Blake B Aleman A Thielscher A Herrmann CS. Interindividual variability of electric fields during transcranial temporal interference stimulation (tTIS). Sci Rep. (2021) 11:20357. 10.1038/s41598-021-99749-0 34645895 PMC8514596

[21] Iszak K Gronemann SM Meyer S Hunold A Zschüntzsch J Bähr M. Why temporal inference stimulation may fail in the human brain: a pilot research study. Biomedicines. (2023) 11:1813. 10.3390/biomedicines11071813 37509455 PMC10376875

[22] Lee S Park J Lee C Im CH. Multipair transcranial temporal interference stimulation for improved focalized stimulation of deep brain regions: a simulation study. Comput Biol Med. (2022) 143:105337. 10.1016/j.compbiomed.2022.105337 35220075

[23] Demchenko I Tailor I Chegini S Yu H Gholamali Nezhad F Rueda A. Human applications of transcranial temporal interference stimulation: a systematic review. medRxiv. 2025-05. (2025). 10.1101/2025.05.16.25327804 40835066

[24] Soroushi H Abbasi S Du Y Ning N Lei Y. Temporal interference stimulation: mechanisms, optimization, validation, and clinical prospects a comprehensive review. Comput Stat. (2025) 17:e70031. 10.1002/wics.70031

[25] Peng J Du Z Piao Y Yu X Huang K Tang Y. Advances in the application of temporal interference stimulation: a scoping review. Front Hum Neurosci. (2025) 19:1536906. 10.3389/fnhum.2025.1536906 40520674 PMC12162641

[26] Arksey H O'malley L. Scoping studies: towards a methodological framework. Int J Soc Res Methodol. (2005) 8:19–32. 10.1080/1364557032000119616

[27] Levac D Colquhoun H O'brien KK. Scoping studies: advancing the methodology. Implement Sci. (2010) 5:69. 10.1186/1748-5908-5-69 20854677 PMC2954944

[28] Sarmiento C San-Juan D Prasath V. Brief history of transcranial direct current stimulation (tDCS): from electric fishes to microcontrollers. Psychol Med. (2016) 46:3259–61. 10.1017/S0033291716001926 27572999

[29] Cambridge NA. Electrical Apparatus Used in Medicine Before 1900. New York: SAGE Publications. (1977). 335397 10.1177/003591577707000909 PMC1543370

[30] Fitzgerald PB. Transcranial pulsed current stimulation: a new way forward? Clin Neurophysiol. (2014) 125:217–9. 10.1016/j.clinph.2013.10.009 24210514

[31] Zheng S Fu T Yan J Zhu C Li L Qian Z. Repetitive temporal interference stimulation improves jump performance but not the postural stability in young healthy males: a randomized controlled trial. J Neuroeng Rehabil. (2024) 21:38. 10.1186/s12984-024-01336-7 38509563 PMC10953232

[32] Wessel MJ Beanato E Popa T Windel F Vassiliadis P Menoud P. Noninvasive theta-burst stimulation of the human striatum enhances striatal activity and motor skill learning. Nat Neurosci. (2023) 26:2005–16. 10.1038/s41593-023-01457-7 37857774 PMC10620076

[33] Ma R Xia X Zhang W Lu Z Wu Q Cui J. High gamma and beta temporal interference stimulation in the human motor cortex improves motor functions. Front Neurosci. (2022) 15:800436. 10.3389/fnins.2021.800436 35046771 PMC8761631

[34] Zhang Y Zhou Z Zhou J Qian Z Lü J Li L. Temporal interference stimulation targeting right frontoparietal areas enhances working memory in healthy individuals. Front Hum Neurosci. (2022) 16:918470. 10.3389/fnhum.2022.918470 36393981 PMC9650295

[35] Zhu Z Xiong Y Chen Y Jiang Y Qian Z Lu J. Temporal interference (TI) stimulation boosts functional connectivity in human motor cortex: a comparison study with transcranial direct current stimulation (tDCS). Neural Plast. (2022) 2022:7605046. 10.1155/2022/7605046 35140781 PMC8820942

[36] von Conta J Kasten FH Ćurčić-Blake B Schellhorn K Herrmann CS. Characterizing low-frequency artifacts during transcranial temporal interference stimulation (tTIS). Neuroimage. (2022) 2:100113. 10.1016/j.ynirp.2022.100113 40567308 PMC12172777

[37] Piao Y Ma R Weng Y Fan C Xia X Zhang W. Safety evaluation of employing temporal interference transcranial alternating current stimulation in human studies. Brain Sci. (2022) 12:1194. 10.3390/brainsci12091194 36138930 PMC9496688

[38] Vassiliadis P Beanato E Popa T Windel F Morishita T Neufeld E. Non-invasive stimulation of the human striatum disrupts reinforcement learning of motor skills. Nat Hum Behav. (2024) 8:1581–98. 10.1038/s41562-024-01901-z 38811696 PMC11343719

[39] Demchenko I Rampersad S Datta A Horn A Churchill NW Kennedy SH. Target engagement of the subgenual anterior cingulate cortex with transcranial temporal interference stimulation in major depressive disorder: a protocol for a randomized sham-controlled trial. Front Neurosci. (2024) 18:1390250. 10.3389/fnins.2024.1390250 39268031 PMC11390435

[40] Yang C Xu Y Du Y Shen X Li T Chen N. Transcranial temporal interference subthalamic stimulation for treating motor symptoms in Parkinson's disease: a pilot study. Brain Stimulat. (2024) 17:1250–2. 10.1016/j.brs.2024.10.012 39486623

[41] Vassiliadis P Stiennon E Windel F Wessel MJ Beanato E Hummel FC. Safety, tolerability and blinding efficiency of non-invasive deep transcranial temporal interference stimulation: first experience from more than 250 sessions. J Neural Eng. (2024) 21:024001. 10.1088/1741-2552/ad2d32 38408385

[42] Fertonani A Ferrari C Miniussi C. What do you feel if I apply transcranial electric stimulation? Safety, sensations and secondary induced effects. Clin Neurophysiol. (2015) 126:2181–8. 10.1016/j.clinph.2015.03.015 25922128

[43] Antal A Alekseichuk I Bikson M Brockmöller J Brunoni AR Chen R. Low intensity transcranial electric stimulation: safety, ethical, legal regulatory and application guidelines. Clin Neurophysiol. (2017) 128:1774–809. 10.1016/j.clinph.2017.06.001 28709880 PMC5985830

[44] Reckow J Rahman-Filipiak A Garcia S Schlaefflin S Calhoun O DaSilva AF. Tolerability and blinding of 4x1 high-definition transcranial direct current stimulation (HD-tDCS) at two and three milliamps. Brain Stimul. (2018) 11:991–7. 10.1016/j.brs.2018.04.022 29784589 PMC6512313

[45] El Jamal C Harrie A Rahman-Filipiak A Iordan AD DaSilva AF Ploutz-Snyder R. Tolerability and blinding of high-definition transcranial direct current stimulation among older adults at intensities of up to 4 mA per electrode. Brain Stimul. (2023) 16:1328–35. 10.1016/j.brs.2023.08.025 37660936 PMC11218548

[46] Gandiga PC Hummel FC Cohen LG. Transcranial DC stimulation (tDCS): a tool for double-blind sham-controlled clinical studies in brain stimulation. Clin Neurophysiol. (2006) 117:845–50. 10.1016/j.clinph.2005.12.003 16427357

[47] Wessel M Zimerman M Timmermann J Hummel F. Eyelid myokymia in an older subject after repetitive sessions of anodal transcranial direct current stimulation. Brain Stimulat. (2013) 6:463–5. 10.1016/j.brs.2012.09.002 23137701

[48] von Conta J Kasten FH Schellhorn K Ćurčić-Blake B Aleman A Herrmann CS. Benchmarking the effects of transcranial temporal interference stimulation (tTIS) in humans. Cortex. (2022) 154:299–310. 10.1016/j.cortex.2022.05.017 35839572

[49] Cassarà AM Newton TH Zhuang K Regel SJ Achermann P Kuster N. Safety recommendations for temporal interference stimulation in the brain. bioRxiv.2022-12. (2022). 10.1101/2022.12.15.520077

[50] Gomez-Tames J Asai A Hirata A. Multiscale computational model reveals nerve response in a mouse model for temporal interference brain stimulation. Front Neurosci. (2021) 15:684465. 10.3389/fnins.2021.684465 34276293 PMC8277927

[51] Mojiri Z Akhavan A Rouhani E Zahabi SJ. Quantitative analysis of noninvasive deep temporal interference stimulation: a simulation and experimental study. Heliyon. (2024) 10:e29482. 10.1016/j.heliyon.2024.e29482 38655334 PMC11035070

[52] Plovie T Schoeters R Tarnaud T Martens L Joseph W Tanghe E. Influence of temporal interference stimulation parameters on point neuron excitability. In: 2022 44th Annual International Conference of the IEEE Engineering in Medicine & *Biology Society (EMBC)*. IEEE (2022). p. 2365–2368. 10.1109/EMBC48229.2022.9871641 36085979

[53] Karimi N Amirfattahi R Zeidaabadi Nezhad A. Neuromodulation effect of temporal interference stimulation based on network computational model. Front Hum Neurosci. (2024) 18:1436205. 10.3389/fnhum.2024.1436205 39386280 PMC11461302

[54] Modak P Fine J Colon B Need E Cheng H Hulvershorn L. Temporal interference electrical neurostimulation at 20 Hz beat frequency leads to increased fMRI BOLD activation in orbitofrontal cortex in humans. Brain Stimul. (2024) 17:867–75. 10.1016/j.brs.2024.07.014 39059712

[55] Vöröslakos M Takeuchi Y Brinyiczki K Zombori T Oliva A Fernández-Ruiz A. Direct effects of transcranial electric stimulation on brain circuits in rats and humans. Nat Commun. (2018) 9:483. 10.1038/s41467-018-02928-3 29396478 PMC5797140

[56] Baek K Park CR Jang S Shim WH Kim YR. Anesthetic modulations dissociate neuroelectric characteristics between sensory-evoked and spontaneous activities across bilateral rat somatosensory cortical laminae. Sci Rep. (2022) 12:11661. 10.1038/s41598-022-13759-0 35804171 PMC9270342

[57] Huang Y Parra LC. Can transcranial electric stimulation with multiple electrodes reach deep targets? Brain Stimul. (2019) 12:30–40. 10.1016/j.brs.2018.09.010 30297323 PMC6301116

[58] Qi S Liu X Yu J Liang Z Liu Y Wang X. Temporally interfering electric fields brain stimulation in primary motor cortex of mice promotes motor skill through enhancing neuroplasticity. Brain Stimul. (2024) 17:245–57. 10.1016/j.brs.2024.02.014 38428583

[59] Howell B McIntyre CC. Feasibility of interferential and pulsed transcranial electrical stimulation for neuromodulation at the human scale. Neuromodulation. (2021) 24:843–53. 10.1111/ner.13137 32147953

[60] Radyt e E Wendt K Sorkhabi MM O'Shea J Denison T. Relative comparison of non-invasive brain stimulation methods for modulating deep brain targets. In: 2022 44th Annual International Conference of the IEEE Engineering in Medicine & *Biology Society (EMBC)*. IEEE (2022). p. 1715–1718. 10.1109/EMBC48229.2022.9871476 36085882 PMC7618923

[61] Huang Y. Visualizing interferential stimulation of human brains. Front Hum Neurosci. (2023) 17:1239114. 10.3389/fnhum.2023.1239114 37954939 PMC10637574

[62] Lee S Lee C Park J Im CH. Individually customized transcranial temporal interference stimulation for focused modulation of deep brain structures: a simulation study with different head models. Sci Rep. (2020) 10:11730. 10.1038/s41598-020-68660-5 32678264 PMC7366675

[63] Huang Y Datta A Parra LC. Optimization of interferential stimulation of the human brain with electrode arrays. J Neural Eng. (2020) 17:036023. 10.1088/1741-2552/ab92b3 32403096

[64] Cao J Grover P. Stimulus: noninvasive dynamic patterns of neurostimulation using spatio-temporal interference. IEEE Trans Biomed Eng. (2019) 67:726–37. 10.1109/TBME.2019.2919912 31150335

[65] Missey F Rusina E Acerbo E Botzanowski B Trébuchon A Bartolomei F. Orientation of temporal interference for non-invasive deep brain stimulation in epilepsy. Front Neurosci. (2021) 15:633988. 10.3389/fnins.2021.633988 34163317 PMC8216218

[66] Honarbakhsh B Mohammadzadeh M. Focusing the temporally interfering electric fields in non-invasive deep brain stimulation. Electron Lett. (2020) 56:1401–3. 10.1049/el.2020.229540795881

[67] Stoupis D Samaras T. Non-invasive stimulation with temporal interference: optimization of the electric field deep in the brain with the use of a genetic algorithm. J Neural Eng. (2022) 19:056018. 10.1088/1741-2552/ac89b3 35970146

[68] Wang M Lou K Liu Z Wei P Liu Q. Multi-objective optimization via evolutionary algorithm (MOVEA) for high-definition transcranial electrical stimulation of the human brain. Neuroimage. (2023) 280:120331. 10.1016/j.neuroimage.2023.120331 37604295

[69] Schmidhuber J. Deep learning in neural networks: an overview. Neural Netw. (2015) 61:85–117. 10.1016/j.neunet.2014.09.003 25462637

[70] Karimi F Attarpour A Amirfattahi R Nezhad AZ. Computational analysis of non-invasive deep brain stimulation based on interfering electric fields. Phys Med Biol. (2019) 64:235010. 10.1088/1361-6560/ab5229 31661678

[71] Bahn S Lee C Kang BY. A Computational Study on the Optimization of Transcranial Temporal Interfering Stimulation With High-Definition Electrodes Using Unsupervised Neural Networks. Hoboken, USA: Wiley Online Library. (2023). 10.1002/hbm.26181 PMC9980883 36527707

[72] Wang H Sun W Zhang J Yan Z Wang C Wang L. Influence of layered skull modeling on the frequency sensitivity and target accuracy in simulations of transcranial current stimulation. Hum Brain Mapp. (2021) 42:5345–56. 10.1002/hbm.25622 34390079 PMC8519867

[73] Khatoun A Asamoah B Mc Laughlin M. Investigating the feasibility of epicranial cortical stimulation using concentric-ring electrodes: a novel minimally invasive neuromodulation method. Front Neurosci. (2019) 13:440965. 10.3389/fnins.2019.00773 31396045 PMC6667561

[74] Ahsan F Chi T Cho R Sheth SA Goodman W Aazhang B. EMvelop stimulation: minimally invasive deep brain stimulation using temporally interfering electromagnetic waves. J Neural Eng. (2022) 19:046005. 10.1088/1741-2552/ac7894 35700717

[75] Lee S Park J Lim S Kwak Y Jang DP Kim DH. Feasibility of epidural temporal interference stimulation for minimally invasive electrical deep brain stimulation: simulation and phantom experimental studies. J Neural Eng. (2022) 19:056003. 10.1088/1741-2552/ac8503 36066021

[76] Luff CE Dzialecka P Acerbo E Williamson A Grossman N. Pulse-width modulated temporal interference (PWM-TI) brain stimulation. Brain Stimul. (2024) 17:92–103. 10.1016/j.brs.2023.12.010 38145754

[77] Kish LB Antal A. Non-invasive deep-brain stimulations by spatio-temporal fourier synthesis. Fluct Noise Lett. (2024) 23:2450049. 10.1142/S0219477524500494

[78] Zhu X Li Y Zheng L Shao B Liu X Li C. Multi-point temporal interference stimulation by using each electrode to carry different frequency currents. IEEE Access. (2019) 7:168839–48. 10.1109/ACCESS.2019.2947857

[79] Khatoun A Asamoah B Mc Laughlin M. A computational modeling study to investigate the use of epicranial electrodes to deliver interferential stimulation to subcortical regions. Front Neurosci. (2021) 15:779271. 10.3389/fnins.2021.779271 34975383 PMC8716464

[80] Levy RM Harvey RL Kissela BM Winstein CJ Lutsep HL Parrish TB. Epidural electrical stimulation for stroke rehabilitation: results of the prospective, multicenter, randomized, single-blinded everest trial. Neurorehabil Neural Repair. (2016) 30:107–19. 10.1177/1545968315575613 25748452

[81] Williams NR Bentzley BS Hopkins T Pannu J Sahlem GL Takacs I. Optimization of epidural cortical stimulation for treatment-resistant depression. Brain Stimul. (2018) 11:239–40. 10.1016/j.brs.2017.09.001 28918944

[82] Brahma T Guillen A Moreno J Datta A Huang Y. On the need of individually optimizing temporal interference stimulation of human brains due to inter-individual variability. Brain Stimul. (2025) 18:1373–1388. 10.1016/j.brs.2025.07.006 40645287 PMC12486182

[83] Yatsuda K Fernández-Corazza M Yu W Gomez-Tames J. Population-optimized electrode montage approximates individualized optimization in transcranial temporal interference stimulation. Comput Biol Med. (2025) 192:110223. 10.1016/j.compbiomed.2025.110223 40286492

[84] Acerbo E Botzanowski B Dellavale D Stern MA Cole E Gutekunst CA. Improved temporal and spatial focality of non-invasive deep-brain stimulation using multipolar single-pulse temporal interference with applications in epilepsy. bioRxiv.024-01. (2024). 10.1101/2024.01.11.575301

[85] Acerbo E Jegou A Luff C Dzialecka P Botzanowski B Missey F. Focal non-invasive deep-brain stimulation with temporal interference for the suppression of epileptic biomarkers. Front Neurosci. (2022) 16:945221. 10.3389/fnins.2022.945221 36061593 PMC9431367

[86] Yang C Xu Y Feng X Wang B Du Y Wang K. Transcranial temporal interference stimulation of the right globus pallidus in Parkinson's disease. Movement Disor. (2024) 40:1061–1069. 10.1002/mds.29967 39133053 PMC12160976

[87] Zhu Z Yin L. A mini-review: recent advancements in temporal interference stimulation in modulating brain function and behavior. Front Hum Neurosci. (2023) 17:1266753. 10.3389/fnhum.2023.1266753 37780965 PMC10539552

